# Orientin Reverses Premature Senescence in Equine Adipose Stromal Cells Affected by Equine Metabolic Syndrome Through Oxidative Stress Modulation

**DOI:** 10.3390/ijms26146867

**Published:** 2025-07-17

**Authors:** Dominika Orzoł, Martyna Kępska, Magdalena Zyzak

**Affiliations:** Department of Experimental Biology, Institute of Biology, Wrocław University of Environmental and Life Sciences, C. K. Norwida 25, 50-375 Wrocław, Poland; 121656@student.upwr.edu.pl (D.O.); martyna.kepska@upwr.edu.pl (M.K.)

**Keywords:** orientin, ASCs, equine metabolic syndrome, senescence, ROS, oxidative stress

## Abstract

Equine metabolic syndrome (EMS) is a prevalent endocrine disorder associated with insulin dysregulation, oxidative stress, and impaired regenerative capacity of adipose-derived stem cells (ASCs). The aim of this study was to evaluate the effects of orientin—a plant-derived flavonoid with known antioxidant properties—on equine ASCs (EqASCs) derived from both clinically healthy and diagnosed EMS-affected mares. EqASCs were treated with orientin to evaluate its biological effects. The analysis included key cellular functions such as proliferative capacity, viability, apoptosis, oxidative stress, senescence, clonogenicity, and migration. Orientin significantly enhanced the proliferative activity of EqASCs, as evidenced by increased Ki67 expression and favorable alterations in cell cycle distribution. In addition, the treatment improved overall cell viability, reduced apoptotic activity, and restored both the clonogenic potential and migratory capacity of the cells, with particularly pronounced effects observed in EqASCs isolated from EMS-affected horses. Importantly, orientin also led to a marked reduction in cellular senescence and oxidative stress, further suggesting its potential as a protective and regenerative agent in metabolically impaired ASCs. These findings indicate that orientin can exert comprehensive cytoprotective effects on EqASCs, with pronounced benefits in cells derived from EMS-affected animals. By improving multiple functional parameters, orientin emerges as a promising candidate for therapeutic strategies aimed at restoring the regenerative potential of ASCs compromised by metabolic dysregulation in horses.

## 1. Introduction

Equine metabolic syndrome (EMS) represents a significant and growing concern within veterinary medicine, affecting an increasing proportion of the global equine population [1]. This endocrine disorder, analogous in many respects to human metabolic syndrome, is defined by a constellation of clinical and metabolic abnormalities, including regional adiposity, increased risk of endocrinopathic laminitis, and insulin dysregulation manifesting as hyperinsulinemia or an exaggerated insulin response [2,3]. First systematically described by Johnson, EMS shares clinical features with peripheral Cushing’s syndrome, particularly in terms of insulin resistance and abnormal fat deposition [4,5]. The pathogenesis of EMS is complex and multifactorial, arising from the interplay of genetic predisposition, inappropriate dietary practices, and lack of physical activity [5,6,7,8]. Emerging evidence has also highlighted the involvement of accelerated cellular aging, or premature senescence, particularly in adipose-derived stromal cells (ASCs), which impairs tissue regeneration and may exacerbate disease progression [5,9].

An emerging and critical dimension of EMS pathophysiology is the progression of premature cellular senescence, a process characterized by irreversible cell cycle arrest accompanied by functional deterioration and altered secretory profiles [10]. Recent evidence suggests that EMS is not merely a metabolic condition but also a disease of accelerated cellular aging, particularly affecting ASCs, which are essential for tissue repair and homeostasis [1,5].

In horses affected by EMS, ASCs exhibit markers of early senescence, including increased expression of senescence-associated β-galactosidase (SA-β-Gal), elevated levels of p53 and p21, and a pro-inflammatory senescence-associated secretory phenotype (SASP) [9]. This cellular phenotype not only limits the regenerative capacity of these progenitor cells but also contributes to a pro-inflammatory microenvironment that exacerbates systemic insulin resistance and local tissue dysfunction. The accumulation of senescent cells within adipose and other metabolic tissues may thus play a pivotal role in the perpetuation and progression of EMS, linking metabolic stress to impaired cellular resilience. Mechanistically, oxidative stress, mitochondrial dysfunction, and chronic low-grade inflammation—common features of EMS—are recognized drivers of premature senescence [1]. The overproduction of reactive oxygen species (ROS) in metabolically stressed adipocytes and stromal cells induces DNA damage responses that activate senescence signaling cascades. Moreover, insulin dysregulation itself may promote senescence by modulating key pathways such as mTOR and FOXO, which regulate cellular metabolism, growth, and longevity [1,11]. Targeting senescence pathways may offer novel therapeutic avenues in the management of EMS. Interventions aimed at reducing oxidative stress, enhancing mitochondrial function, or selectively eliminating senescent cells (senolytics) are under investigation in both human and veterinary contexts and hold promise for restoring metabolic balance and cellular vitality in affected horses [5,9].

Given the central role of premature cellular senescence in the pathogenesis EMS, the identification of bioactive compounds capable of mitigating this process has garnered increasing scientific interest. Among such compounds, orientin, a flavonoid glycoside belonging to the flavone subclass and commonly found in plants such as *Ocimum sanctum* (holy basil) and *Passiflora edulis*, has demonstrated significant antioxidant, anti-inflammatory, and cytoprotective properties that position it as a promising candidate for combating cellular aging in metabolic disorders [5,12].

Recent preclinical studies have shown that orientin attenuates oxidative stress-induced senescence in various cell types by scavenging reactive oxygen species (ROS) and enhancing endogenous antioxidant defenses, such as superoxide dismutase (SOD) and glutathione peroxidase (GPx) [13]. This is particularly relevant in the context of EMS, where elevated oxidative stress within adipose tissue and ASCs contributes to DNA damage and mitochondrial dysfunction—two principal triggers of senescence [9,14,15,16]. At the molecular level, orientin exerts regulatory effects on key signaling pathways associated with longevity and metabolic homeostasis, including the PI3K/Akt, AMPK, and FOXO signaling axes. By modulating of these cascades, orientin supports mitochondrial homeostasis, inhibits of pro-senescent gene expression (such as *p16INK4a* and *p21*), and enhances autophagic flux, thereby promoting cellular resilience and extending the functional lifespan of progenitor cells [5,17]. Furthermore, orientin has been shown to suppress the senescence-associated secretory phenotype (SASP), leading to reduced secretion of inflammatory cytokines like IL-6 and TNF-α, which are known to exacerbate systemic inflammation and insulin resistance in EMS [9,18,19]. Additionally, orientin’s favorable pharmacological profile—including low toxicity, good bioavailability, and the ability to cross cellular membranes—further supports its therapeutic potential [9,20]. Although most existing data are derived from in vitro and rodent studies, its mechanism of action aligns with the major pathological processes identified in EMS, warranting translational investigation in equine models.

This study aims to explore the potential of orientin as a therapeutic agent for EMS, focusing on its protective effects against oxidative stress and premature senescence in EqASCs. Given its pleiotropic actions, we hypothesized that orientin may attenuate the progression of EMS-related cellular impairments and contribute to restoring homeostasis in metabolically compromised stromal cells. Further research is needed to validate these effects in vivo and assess their clinical relevance in equine practice.

## 2. Results

### 2.1. Evaluation of the Cytotoxicity and Biocompatibility of Orientin and GI_50_ Determination

**Figure 1:**
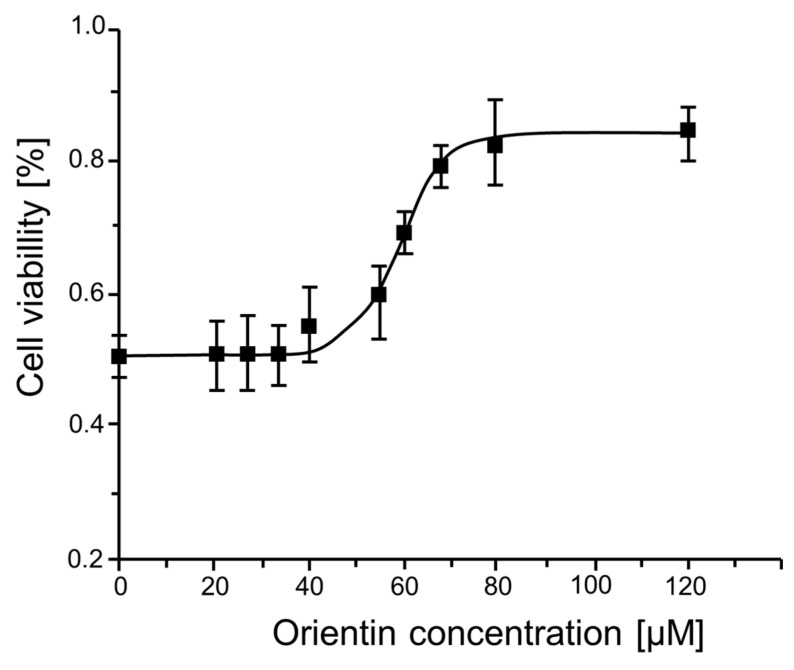
Effect of orientin on equine ASCs viability. Dose-response curve showing cell viability (%) in relation to increasing concentrations of orientin (15 to 130 µM). Data represent mean ± SD from three independent experiments.

To assess the cytotoxicity and biocompatibility of orientin on ASCs after 24 h of treatment with increasing orientin concentrations, the MTT assay was performed. The dose-response curve demonstrated a sigmoidal relationship between orientin concentration (μM) and cell viability (%), with a progressive increase in cell viability observed up to approximately 60 µM, after which the curve plateaus, indicating a saturation effect (Figure 1).

Nonlinear regression analysis was conducted to determine the GI_50_ value (the concentration at which cell viability reaches 50% of the maximal response), which was calculated to be approximately 60 μM. This concentration was subsequently selected for further experiments. The fit of the model is statistically significant (*p* < 0.0001), and the high R^2^ value (close to 0.99) confirms a strong correlation between orientin concentration and cell viability (Supplementary Material).

### 2.2. Orientin Restores Proliferative Capacity and Promotes Cell Cycle Re-Entry in EMS-Derived EqASCs

**Figure 2:**
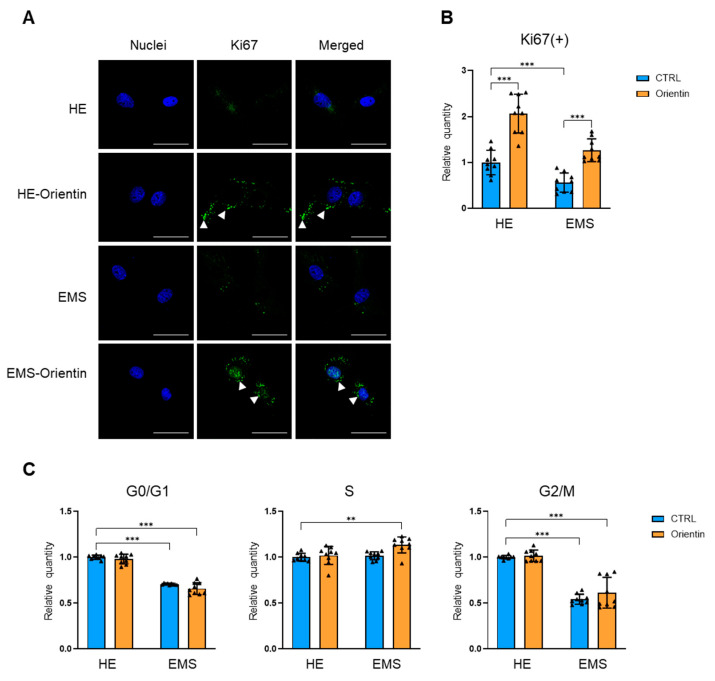
Orientin promotes proliferation and cell cycle progression in EqASCs from EMS-affected horses. (**A**) Representative confocal photomicrographs showing immunofluorescence staining of Ki67 protein (green) and DAPI nuclear counterstaining (blue) in ASCs derived from healthy (HE) and EMS horses, with or without orientin treatment. White arrowheads indicate additional intense Ki67 expression after orientin treatment. Scale bar: 30 µm. (**B**) Quantification of Ki67-positive cells shows a significant increase in proliferative activity following orientin treatment. (**C**) Histograms depicting the distribution of experimental cells across cell cycle stages. Data are presented as mean ± SD, with individual data points shown as triangles; ** *p* < 0.01., *** *p* < 0.001.

Nuclear Ki67 immunostaining revealed significantly reduced proliferative activity in EqASCs derived from EMS-affected horses compared to healthy controls. Ki67 staining showed that EMS cells treated with orientin had a stronger signal compared to the EMS control group (Figure 2A) Quantitative analysis confirmed that orientin significantly increased the proliferation rate compared to EMS cells (Figure 2B; *p* < 0.001). Interestingly, orientin application to control, healthy cells showed similar potentiation of cell proliferation (Figure 2A,B; *p* < 0.001). To assess whether orientin influences cell cycle dynamics, microcapillary flow cytometry-based analysis was performed to establish the differential distribution of EqASCs across G0/G1, S, and G2/M cell cycle phases (Figure 2C). In the G0/G1 stage, a significantly lower proportion of EMS-derived EqASCs was observed compared to healthy controls (*p* < 0.001). In contrast, orientin did not significantly alter the G0/G1 distribution in both healthy and EMS ASCs cells (Figure 2C). In the S phase, orientin markedly increased the proportion of EMS-EqASCs undergoing DNA replication compared to native cells (*p* < 0.01), but no significant changes were observed in G2/M phase entry upon orientin treatment compared to healthy cells with orientin treatment and EMS and orientin-treated cells (Figure 2C).

### 2.3. Orientin Attenuates Cellular Senescence and Enhances Clonogenic and Migratory Potential in EMS-Derived EqASCs

**Figure 3:**
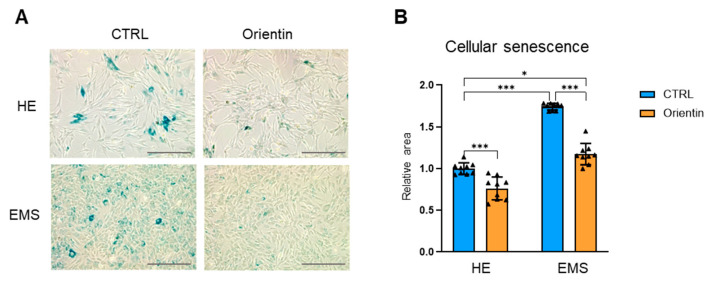
Orientin reduces cellular senescence of EMS-derived EqASCs. (**A**) Representative images of senescence-associated β-galactosidase (SA-β-gal) staining in EqASCs from healthy (HE) and EMS horses, with or without orientin treatment. Blue-stained cells indicate a senescent phenotype. Scale bar: 100 µm. (**B**) Quantification of SA-β-gal-positive cells. Data are presented as mean ± SD, with individual data points shown as triangles; * *p* < 0.05, *** *p* < 0.001.

Senescence-associated β-galactosidase (SA-β-gal) staining was performed to assess the extent of cellular aging in healthy and EMS EqASCs cells and following orientin treatment (Figure 3A). As illustrated in Figure 3A, EMS-EqASCs displayed a pronounced increase in the number of SA-β-gal-positive cells. In contrast, both control and EMS-affected EqASCs treated with orientin exhibited a marked reduction in senescence-associated staining (Figure 3A). Quantitative analysis (Figure 3B) confirmed a significant decrease in the percentage of SA-β-gal-positive cells upon orientin treatment in the healthy (*p* < 0.001) and EMS group (*p* < 0.001), rejuvenating the senescence profile toward levels comparable with healthy controls, even though the level remained significantly higher (*p* < 0.05).

**Figure 4:**
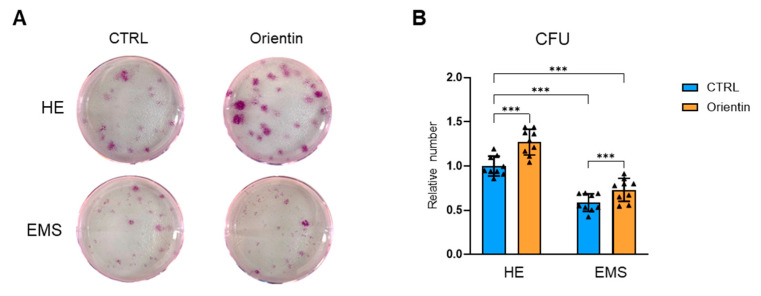
Orientin enhances the clonogenic potential of EMS-derived EqASCs. (**A**) Representative pararosaniline-stained plates from colony-forming unit fibroblast (CFU) assays and (**B**) quantification of colony number in healthy (HE) and EMS-derived EqASCs before (CTRL) and after orientin treatment. Data are presented as mean ± SD, with individual data points shown as triangles; *** *p* < 0.001.

To further investigate the functional impact of orientin on the regenerative potential of aged EqASCs, a colony-forming unit (CFU) assay was conducted. The representative images (Figure 4A) highlight that EMS-derived EqASCs exhibited reduced clonogenic capacity. Treatment with orientin significantly increased the number and size of colonies in both native and EMS-EqASCs cells, as evidenced by pararosaniline staining. Quantification (Figure 4B) revealed a statistically significant increase in clonogenicity following orientin treatment in healthy (*p* < 0.001) and EMS (*p* < 0.001) compared to the untreated cells.

**Figure 5:**
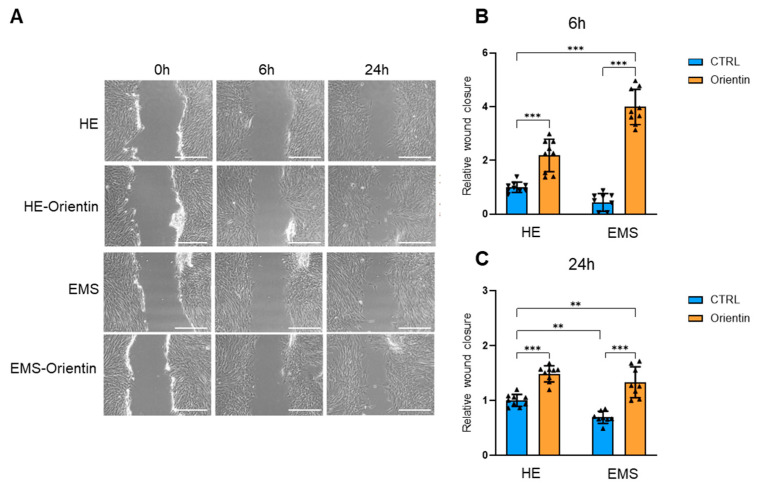
Orientin enhances the migratory potential of EMS-derived EqASCs. (**A**) Representative images of wound healing assay at 0, 6, and 24 h after scratch. Scale bar: 400 µm. (**B**) Quantification of wound closure after 6 and (**C**) 24 h. Data are presented as mean ± SD, with individual data points shown as triangles; ** *p* < 0.01, *** *p* < 0.001.

Cell migration is a key feature of stromal cell regenerative performance and tissue repair. A wound healing (scratch) assay was therefore performed to examine the migratory capacity of EqASCs in response to orientin (Figure 5A). Untreated EMS-EqASCs cells displayed limited migration into the scratch area after over 24 h post-wound induction compared to the native cells (*p* < 0.01). Conversely, orientin-treated cells showed pronounced closure of the wound area, especially after 6 h (Figure 5A,B) in both healthy (*p* < 0.001) and EMS (*p* < 0.001) cells, leading to a higher efficiency of this process in EMS cells compared to native cells (*p* < 0.001). After 24 h (Figure 5A,C), a significant increase in wound closure was similarly observed in both orientin-treated healthy (*p* < 0.001) and EMS groups (*p* < 0.001). However, orientin-treated EMS cells were still characterized by reduced migration rates when compared to healthy ASCs (*p* < 0.01).

### 2.4. Orientin Reduces Apoptosis and Modulates the Expression of Key Apoptotic and Cell Cycle Regulatory Genes in EMS-EqASCs

**Figure 6:**
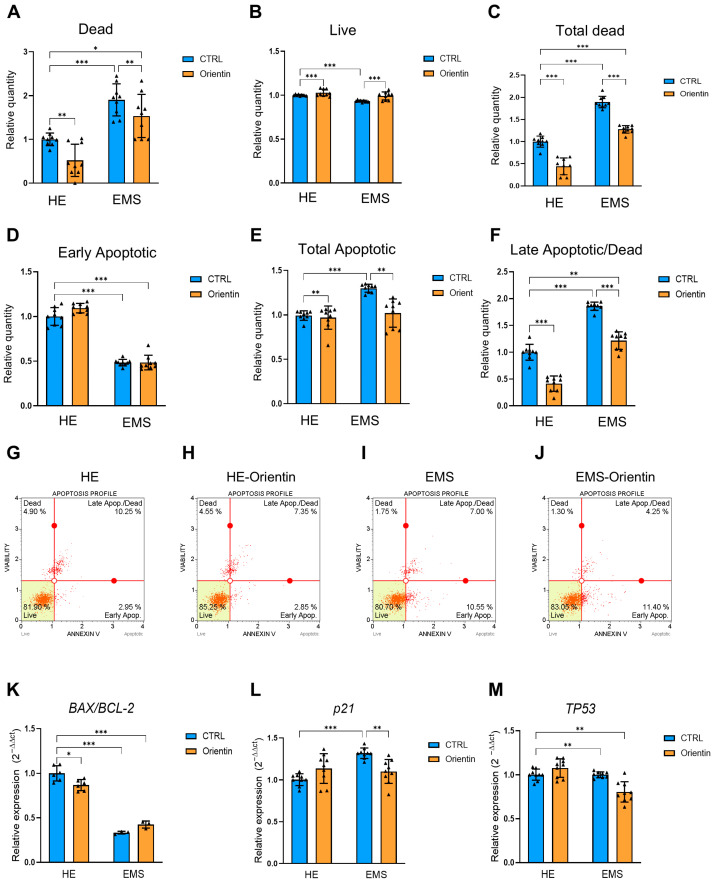
Orientin attenuates apoptosis and modulates the expression of key apoptotic and senescence-related genes in EMS-derived EqASCs. Quantification of (**A**–**C**) live and dead cells and (**D**–**F**) apoptotic healthy (HE) and EMS-derived EqASCs cells. (**G**–**J**) Representative plots from MUSE^®^ Cell Analyzer. (**K**–**M**) Gene expression analysis of *BAX/BCL-2* ratio, *p21*, and *TP53*. Data are expressed as mean ± SD, with individual data points shown as triangles; * *p* < 0.05, ** *p* < 0.01, *** *p* < 0.001.

To assess the pro-survival effects of orientin in EqASCs cells obtained from healthy and EMS horses, cell viability was quantified using the Muse^®^ Annexin V & Dead Cell assay. As shown in Figure 6A–C, EMS-derived EqASCs exhibited a significantly increased proportion of dead (Figure 6A; *p* < 0.001) and total dead (Figure 6C; *p* < 0.001) cells compared to healthy controls and a lower proportion of live cells (Figure 6B; *p* < 0.001). Treatment with orientin led to a marked reduction in the number of dead (Figure 6A) cells in both the healthy (*p* < 0.001) and EMS group (*p* < 0.001). In correlation with this result, an increase in viable cells was noted after orientin treatment (Figure 6B), both in native (*p* < 0.001) and EMS (*p* < 0.001) cells relative to untreated cells.

To further validate the protective effect of orientin against cell death, a detailed quantitative analysis of cell distribution across various apoptosis stages was performed. (Figure 6D–J). The analysis distinguished between early apoptotic, late apoptotic, and live cell populations. Figure 6D shows a significant decrease in early apoptotic cells in EqASCs derived from EMS horses compared to healthy controls (*p* < 0.001). Importantly, treatment with orientin did not reduce the proportion of early apoptotic cells in both healthy and EMS-derived cells. EMS-EqASCs also displayed a markedly elevated number of total apoptotic cells compared to healthy cells (*p* < 0.001). Upon orientin treatment, this population was significantly decreased in both healthy (*p* < 0.01) and EMS-EqASCs (*p* < 0.01) cells, further supporting the compound’s anti-apoptotic efficacy under metabolic stress conditions. Next, a significant increase in the late apoptotic/dead ratio (Figure 6F) of cells in the EMS group compared to healthy controls (*p* < 0.001) was observed. Noteworthy, orientin treatment significantly restored cell viability in healthy (*p* < 0.001) and EMS-EqASCs (*p* < 0.001) cells and also moderately increased viability in EMS orientin-treated cells compared to healthy cells (*p* < 0.01).

Then, changes in the expression levels of key genes involved in apoptosis regulation were analyzed. The expression ratio of *BAX* to *BCL-2* (Figure 6K), a key indicator of the pro-apoptotic to anti-apoptotic signaling balance, was significantly reduced in healthy EqASCs cells following orientin treatment (*p* < 0.05). In contrast, EMS-derived EqASCs exhibited an overall lower basal *BAX/BCL-2* ratio compared to healthy controls (*p* < 0.001. Importantly, orientin treatment did not significantly alter the *BAX/BCL-2* expression in EMS-EqASCs. Gene expression analysis revealed that *p21* mRNA levels (Figure 6L) were significantly elevated in EqASCs derived from EMS horses compared to healthy controls (*p* < 0.001). Treatment with orientin resulted in a significant reduction in *p21* expression only in EMS-derived EqASCs (*p* < 0.01). *TP53*, a central regulator of the DNA damage response and cellular senescence, showed significantly elevated expression in EMS-derived EqASCs compared to healthy controls (Figure 6M *p* < 0.001). In healthy ASCs, orientin treatment did not significantly alter *TP53* expression. Conversely, orientin treatment significantly reduced *TP53* expression in EMS-derived EqASCs (*p* < 0.01) compared to the healthy cells.

### 2.5. Orientin Reduces Oxidative Stress in EMS-Derived EqASCs

Oxidative stress in EqASCs was assessed using MUSE-based analysis of ROS-positive and ROS-negative cell populations, CM-H2DCFDA fluorescence imaging, flow cytometry, and gene expression analysis of key antioxidant enzymes.

**Figure 7:**
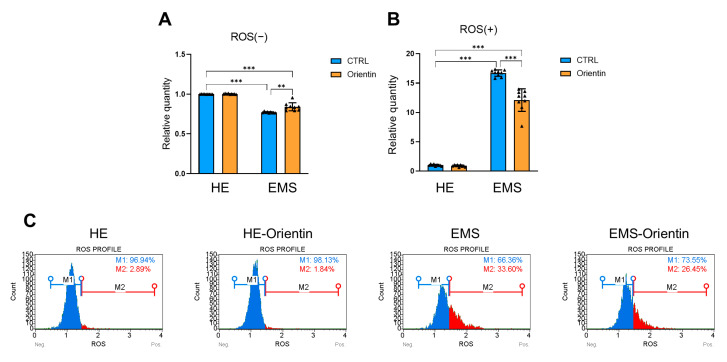
Orientin reduces oxidative stress in EMS-derived EqASCs. (**A**–**C**) Analysis of ROS-positive and ROS-negative cell populations using the MUSE^®^ Cell Analyzer in healthy (HE) and EMS-derived EqASCs. Data are expressed as mean ± SD, with individual data points shown as triangles; ** *p* < 0.01, *** *p* < 0.001.

Microfluidic flow cytometry (Figure 7A–C) showed a significantly lowered number of ROS-negative cells (Figure 7A; *p* < 0.001) and a substantially higher number of ROS-positive cells (Figure 7B; *p* < 0.001) in EqASCs isolated from horses with EMS compared to healthy controls. Orientin treatment significantly increased the ROS-negative population in EMS-derived EqASCs (*p* < 0.01), and decreased the intracellular accumulation of ROS in treated cells (*p* < 0.001).

**Figure 8:**
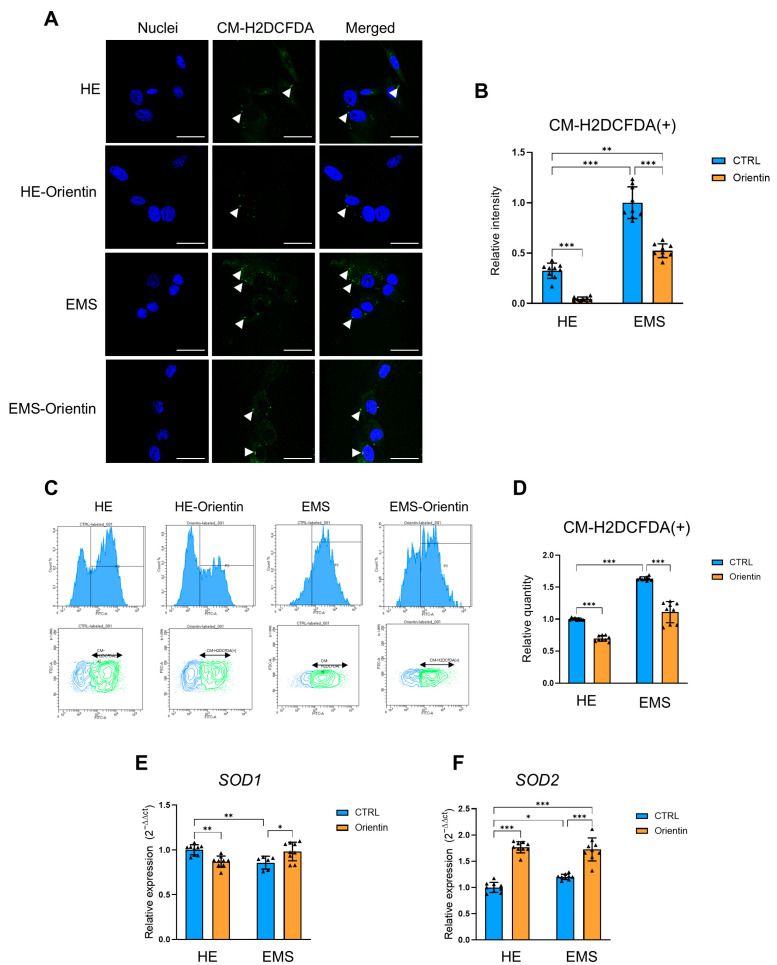
Orientin reduces oxidative stress in EMS-derived EqASCs. (**A**) Representative fluorescence images of ROS accumulation in healthy (HE) and EMS-derived cells, before and after orientin treatment, detected by CM-H2DCFDA staining; green—ROS, blue—DAPI. White arrowheads indicate additional intense CM-H2DCFDA expression before and after orientin treatment. Scale bar: 30 µm. (**B**) Quantification of ROS fluorescence intensity from (**A**). (**C**,**D**) Flow cytometric analysis of ROS levels using CM-H2DCFDA probe. (**E**,**F**) Relative mRNA expression levels of *SOD1* and *SOD2* determined by qPCR. Data are expressed as mean ± SD, with individual data points shown as triangles; * *p* < 0.05, ** *p* < 0.01, *** *p* < 0.001.

The elevated oxidative status in EMS-ASCs was further confirmed by fluorescence microscopy using the CM-H2DCFDA probe (Figure 8A,B). EMS cells showed an intense fluorescence signal indicating ROS accumulation, which was significantly reduced after orientin treatment (Figure 8A). Quantitative analysis (Figure 8B) confirmed a significant increase in the relative fluorescence intensity in the group of EMS-derived EqASCs cells compared to native cells (*p* < 0.001). Importantly, treatment of both groups reduced fluorescence levels in both healthy (*p* < 0.001) and EMS (*p* < 0.001) cells. However, EMS EqASCs treated with orientin presented a consistently higher ROS signal relative to control EqASCs (*p* < 0.01). Flow cytometry analysis using CM-H2DCFDA (Figure 8C,D) corroborated these findings, showing a significantly higher mean fluorescence intensity in EMS cells (*p* < 0.001), which was effectively attenuated by orientin treatment (*p* < 0.05). Orientin treatment reduced this level in both EMS (*p* < 0.001) and native (*p* < 0.001) cells.

To investigate the underlying molecular mechanisms by which orientin influences cellular redox status, the expression of key antioxidant genes *SOD1* and *SOD2* was analyzed (Figure 8E,F). EMS-derived EqASCs showed decreased expression of *SOD1* (Figure 8E; *p* < 0.01) mRNA level compared to healthy cells. Orientin treatment significantly decreased *SOD1* expression in healthy cells (Figure 8E; *p* < 0.01), whereas, on the contrary, it increased the expression of this gene in EMS cells (*p* < 0.5). The expression level of the *SOD2* mRNA was increased in EMS cells (Figure 8F; *p* < 0.05). Orientin treatment additionally increased this level in both healthy (Figure 8F; *p* < 0.001) and EMS-derived cells (*p* < 0.001).

## 3. Discussion

This study provided compelling evidence that orientin, a flavonoid glycoside, exerts multifaceted cytoprotective effects on EqASCs, particularly those derived from horses suffering from equine metabolic syndrome (EMS). The compound appears to significantly influence several key cellular mechanisms, including modulation of apoptosis, enhancement of cell cycle progress, attenuation of oxidative stress, and restoration of regenerative capacity. These properties highlighted orientin’s potential as a supportive agent in regenerative therapies, especially in cases where stromal cell function was compromised due to metabolic dysfunctions.

The results of the present study demonstrated that orientin significantly enhanced Ki67 expression and increased the number of cells in the S phase of the cell cycle in EqASCs obtained from horses affected by EMS. Ki67 is a well-established marker of cellular proliferation, expressed during the active phases of the cell cycle (G1, S, G2, and M), but absent in quiescent (G0) cells, making it a reliable indicator of proliferative activity [21,22]. The observed increase in S-phase cells suggests that orientin facilitated the transition from the G1 to S phase, potentially through modulation of cyclin-dependent kinases (CDKs) and their associated cyclins. Previous studies showed that orientin can induce G0/G1 cell cycle arrest in human colorectal carcinoma HT29 cells by upregulating the CDK inhibitor p21^WAF1/CIP1^ and downregulating cyclins D1 and E, as well as CDK2 and CDK4 [23]. Although our study focused on EqASCs rather than cancer cells, it is plausible that orientin exerts similar regulatory effects on the cell cycle machinery in EqASCs. Interestingly, despite the pronounced increase in S phase cells, no significant change was observed in the G2/M cell population following orientin treatment. This apparent discrepancy may be explained by the relatively short incubation period of 24 h. The population doubling time of EqASCs in vitro typically ranges from 48 to 72 h, and in cells derived from metabolically compromised animals, such as those with EMS, this time might have been even longer due to senescent-like features and decreased metabolic activity [24,25]. Consequently, the cells may not have had sufficient time to progress into mitosis during the experimental window, although the observed increase in S phase cells strongly indicated preparation for cell cycle entry and active DNA replication.

The observed enhancement of clonogenic potential in both healthy and EMS-derived ASCs following orientin treatment underscored its role in promoting self-renewal and stemness. Clonogenic assays, which evaluated a cell’s capacity for unlimited division, were pivotal in assessing stem cell functionality. The increase in both the number and size of colonies suggests that orientin might have activated signaling pathways supporting stem cell maintenance and proliferation. Several key signaling pathways are known to regulate stem cell self-renewal and clonogenicity. For instance, the Wnt/β-catenin pathway is integral in maintaining the undifferentiated state and promoting proliferation in various stem cell types [26]. Inhibition of Wnt signaling was shown to reduce colony formation in human embryonic stem cells [27], highlighting its role in self-renewal processes. Similarly, the PI3K/Akt pathway is crucial for stem cell survival and proliferation, with studies demonstrating that its activation supports the self-renewal of embryonic stem cells [28,29]. Moreover, the JNK signaling pathway was implicated in regulating the traits of various stem cells, including their self-renewal and differentiation capacities [30]. Activation of these pathways might have underlain the observed effects of orientin on ASC clonogenicity. Phytochemicals, such as orientin, were recognized for their potential to modulate these signaling pathways [20,31]. For example, certain flavonoids can enhance stem cell proliferation and counteract senescence by influencing pathways like Wnt/β-catenin and PI3K/Akt [32]. Therefore, the effects of orientin on EqASCs clonogenicity might have been attributed to its capacity to modulate these critical signaling cascades, thereby promoting stem cell self-renewal and proliferation. These findings provided a basis for further investigation into orientin’s therapeutic potential in regenerative medicine.

The observed enhancement in migratory capacity of orientin-treated ASCs, as evidenced by accelerated wound closure in scratch assays, indicated improved cell motility and potential for tissue repair. Cell migration is a critical component of tissue re-generation, involving intricate coordination of cytoskeletal dynamics and interactions with the extracellular matrix. Orientin, a flavonoid compound, was shown to modulate signaling pathways associated with cell migration [33]. In MCF-7 breast cancer cells, orientin inhibited migration and invasion by suppressing the PKCα/ERK/AP-1/STAT3 signaling axis, leading to decreased expression of matrix metalloproteinase-9 (MMP-9) and interleukin-8 (IL-8) [34]. These findings suggest that orientin can influence key pathways governing cell motility. Furthermore, orientin has demonstrated the ability to inhibit the MAPK signaling pathway, which plays a pivotal role in regulating cell migration. In studies involving fibroblast-like synovial cells from rheumatoid arthritis patients, orientin treatment resulted in reduced phosphorylation of ERK, JNK, and p38 MAPKs, correlating with decreased migratory and invasive behaviors [33]. In addition to its effects on signaling pathways, orientin was reported to promote wound healing processes [35]. In 3T3 fibroblast cells, orientin enhanced cell proliferation and migration, contributing to improved wound closure in vitro [36]. Similarly, isoorientin, a structurally related compound, facilitated excisional skin wound healing in mice by modulating inflammatory responses and promoting tissue remodeling [35,36]. Collectively, these findings support the notion that orientin can also affect the EqASCs’ migratory capacity through modulation of key signaling pathways and promotion of wound healing mechanisms, but further investigations are warranted to elucidate the precise molecular mechanisms underlying orientin’s effects on EqASCs migration and to explore its potential therapeutic applications in regenerative medicine.

Mesenchymal stromal cells (MSCs), including ASCs, are increasingly utilized in both human and veterinary regenerative medicine owing to their multipotency, paracrine activity, and immunomodulatory capacity [37,38]. However, in metabolically dysregulated environments such as EMS, the therapeutic efficacy of ASCs may be impaired due to increased levels of oxidative stress, mitochondrial dysfunction, and apoptosis [39]. Therefore, strategies aimed at preserving ASCs viability and functionality in such pathophysiological states are of paramount importance. One of the most striking findings of this study was orientin’s capacity to modulate apoptosis in both healthy and EMS-affected EqASCs. The observed reduction in the BAX/BCL-2 expression ratio indicates a shift in the cellular equilibrium towards survival, suggesting that orientin may inhibit the intrinsic apoptotic pathway [23]. This shift is particularly beneficial in EMS-ASCs, where elevated pro-apoptotic signaling contributed to the depletion of the stromal cell pool and a subsequent decline in regenerative performance. Previous studies have confirmed that orientin exerts anti-apoptotic effects via mitochondrial pathways, notably through suppression of cytochrome c release and inhibition of caspase-3 activation. In models of oxidative and metabolic stress, orientin has been shown to increase *BCL-2* expression while downregulating *BAX*, thereby stabilizing mitochondrial membranes and reducing the loss of mitochondrial potential [40]. Furthermore, orientin can modulate the PI3K/Akt pathway, which plays a key role in regulating cell survival and response to oxidative stress [41]. In the context of endothelial cells, orientin may affect the activation of the PI3K/Akt/eNOS pathway, leading to increased nitric oxide (NO) production and improved endothelial function. Although direct studies on the effects of orientin on endothelial cells are limited, there is evidence for its beneficial effects in the context of oxidative stress and apoptosis in other cell types [42].

Oxidative stress is a key pathophysiological factor in EMS, contributing to cellular dysfunction, accelerated senescence, and impaired regenerative capacity of stromal cells. In our study, we observed that orientin, a natural flavonoid, significantly reduced the levels of reactive oxygen species (ROS) in EqASCs from EMS-affected horses (EMS-EqASCs), indicating its potent antioxidant properties. The mechanism of action of orientin involves the activation of the Nrf2/HO-1 signaling pathway, which plays a crucial role in regulating the cellular antioxidant response [40]. In models of oxidative injury, orientin promoted the translocation of the transcription factor Nrf2 into the nucleus, leading to increased expression of antioxidant enzymes such as heme oxygenase-1 (HO-1), NAD(P)H quinone dehydrogenase 1 (NQO1), and glutamate–cysteine ligase catalytic subunit (GCLC). This cascade ultimately reduces oxidative stress and protects cells from damage [43]. Furthermore, orientin was shown to enhance the activity of superoxide dismutase (SOD) enzymes, including both cytoplasmic SOD1 and mitochondrial SOD2. The upregulation of SOD2 is particularly important, as this enzyme plays a critical role in neutralizing superoxide anions within mitochondria, thereby safeguarding cells against oxidative injury [44]. In addition, orientin exhibited protective effects in various models of oxidative damage, including liver injury induced by d-galactosamine and lipopolysaccharide (d-GalN/LPS). In these models, orientin activated the Nrf2/Keap1 axis, resulting in decreased serum levels of liver enzymes ALT and AST and improved liver histopathology [45].

Reduction in senescence-associated β-galactosidase (SA-β-gal) staining in orientin-treated ASCs suggests a reversal of senescent phenotypes. Cellular senescence, characterized by irreversible cell cycle arrest, is a hallmark of aging and metabolic disorders, often leading to diminished regenerative capacity [1]. The ability of orientin to attenuate senescence markers in our studies indicates its potential in rejuvenating aged or metabolically compromised cells. In the context of senescence, orientin has been reported to possess antioxidant properties that mitigate cellular aging processes. Previous studies have demonstrated that orientin can reduce oxidative stress by scavenging free radicals and enhancing the activity of antioxidant enzymes. For instance, in D-galactose-induced aging models, orientin administration improved antioxidant enzyme levels and reduced markers of oxidative damage, thereby delaying aging-related changes [13,46]. Furthermore, orientin was shown to improve mitochondrial function, which is closely linked to cellular senescence [20]. Mitochondrial dysfunction is a key contributor to the onset of senescence, as it leads to increased production of reactive oxygen species (ROS) and subsequent cellular damage. By enhancing mitochondrial homeostasis, orientin might have alleviated senescence and promote cellular longevity. Recent research indicated that orientin can improve mitochondrial function and inhibit chondrocyte senescence via the PI3K/AKT/NF-κB signaling pathway [20]. In summary, the anti-senescent effects of orientin in ASCs may be mediated through its antioxidant properties and its ability to enhance mitochondrial function. These findings suggest that orientin holds promise as a therapeutic agent for rejuvenating aged or metabolically compromised stem cells, thereby improving their regenerative potential.

While this study provides evidence for the cytoprotective and anti-apoptotic effects of orientin in ASCs under metabolic stress, it is important to note that the findings are based solely on in vitro cellular models. The absence of in vivo validation represents a limitation that must be addressed in future research. Cellular systems, although useful for mechanistic insight, do not fully replicate the complex physiological and metabolic environment present in living organisms, particularly in the context of metabolic syndrome and its systemic effects. Therefore, to better understand the therapeutic potential and biological behavior of orientin, future studies should incorporate animal models of EMS or comparable mammalian models that simulate the metabolic, inflammatory, and oxidative stress conditions observed in the disease. Such studies would allow for assessment of orientin’s bioavailability, pharmacokinetics, tissue-specific effects, and long-term safety profile. Moreover, investigating orientin’s influence on systemic oxidative stress, insulin sensitivity, inflammatory markers, and adipose tissue remodeling in vivo would provide critical translational insight and support the development of orientin-based therapies for metabolic disorders.

## 4. Materials and Methods

### 4.1. Equine ASC Cell Culture and Orientin Treatment

Healthy and EMS-affected Equine ASCs were obtained from the cell collection of the Department of Experimental Biology, University of Environmental and Life Sciences, Wrocław, Poland. ASCs cultures were maintained in 75 cm^2^ flasks and cultured in Dulbecco’s modified Eagle’s medium (DMEM) containing 1000 mg/L glucose (Life Technologies, Paisley, UK), supplemented with 5% of fetal bovine serum (FBS; Sigma-Aldrich, St. Louis, MO, USA), and 1% of a penicillin and streptomycin solution (PS; Sigma-Aldrich, St. Louis, MO, USA), and incubated at 37 °C in a humidified 5% CO_2_, 95% air atmosphere. Cultured cells were harvested every three days (80–90% of confluence) and detached from the flasks with a trypsin–EDTA solution (Sigma-Aldrich, St. Louis, MO, USA). The media were changed every second day.

The orientin was purchased from Cayman Chemicals (Ann Arbor, MI, USA) in the form of a crystalline solid and for this study it was dissolved in a sodium hydroxide solution (NaOH). After the determination of cytotoxic activity of orientin, described below, the cells were treated with orientin with the final concentration of 60 μM under standard conditions for 24 h before testing.

For each experimental method, data were obtained from three independent biological replicates (cells isolated from three healthy and three EMS horses), each analyzed in at least three technical replicates. For most methods, technical replicates were performed in separate wells, except for assays using microscopy techniques, where technical replicates referred to different fields of view on the same sample.

### 4.2. Determination of Cytotoxic Activity of Orientin

The determination of cytotoxic activity of orientin was examined by the MTT assay [47]. The MTT assay was performed in a 96-well flat-bottomed plate; 200 μL of culture medium containing 0.2 × 10^5^ cells was distributed among the wells and incubated for 24 h. The culture medium was then removed, washed with DPBS solution (Sigma-Aldrich, St. Louis, MO, USA), and 200 μL each of fresh medium with the appropriate concentration of orientin was added. The effect of orientin on EqASCs was studied at successive concentrations of 15, 45, 50, 60, 70, 80, 100, 120, and 130 µM. A solution of NaOH (sodium hydroxide) was added to one of the wells as a control. Cells were incubated for 24 h under standard conditions.

After incubation, the medium with orientin was removed and 200 µL of culture medium with MTT reagent (Sigma-Aldrich, St. Louis, MO, USA) at a concentration of 5 mg/mL was added to each well. The cell culture was carried out for 2 h under the same standard conditions. The solution was removed from the wells, after which 100 µL of DMSO solution (AppliChem, Darmstadt, Germany) was added to each well and incubated for 20 min. Absorbance measurements of solutions with dissolved formazan were performed spectrophotometrically at 570 nm using a Synergy H1 plate reader (BioTek^®^ Instruments, Inc., Winooski, VT, USA).

### 4.3. Evaluation of Oxidative Stress

(a) Quantification of ROS accumulation using Muse^®^ Oxidative Stress assay

The reactive oxygen species accumulation (ROS) was measured using the Muse^®^ Oxidative Stress Assay Kit (Luminex, Austin, TX, USA) according to the manufacturer’s protocol. After trypsinization, 200 µL of Muse Oxidative Stress Working Solution was added to the cell pellet and incubated 30 min at 37 °C. Then, the ROS-negative and ROS-positive cells were counted using a Muse™ Cell Analyzer (EMD Millipore, Temecula, CA, USA).

(b) Fluorescence microscopy-based detection of intracellular ROS with CM-H_2_DCFDA

Furthermore, the reactive oxygen species were stained in cells using the CM-H2DCFDA fluorescent dye (General Oxidative Stress Indicator) (Invitrogen Life Technologies, Eugene, OR, USA). The cells were stained according to manufacturers’ instructions. Four hundred microliters of medium containing 0.6 × 10^5^ EqASCs were seeded on coverslips in a 24-well plate. After treating the cells with orientin for 24 h, they were washed with DPBS and then CM-H2DCFDA staining solution (10 µM, prepared in culture medium without FBS and without PS) was added. The cells were then incubated for 30 min in an incubator, and after washing again with DPBS, they were fixed in 4% PFA (Sigma-Aldrich, St. Louis, MO, USA) for 15 min at room temperature (RT). Afterward, they were washed with DPBS three times and the slides were closed using the Fluoroshield™ with DAPI (Sigma-Aldrich, St. Louis, MO, USA). Microscopic preparations were observed using a Leica DMi8 (Leica Microsystems, Wetzlar, Germany) confocal microscope with a magnification of ×630. The photomicrographs were finally analyzed in Fiji using ImageJ Software (version 1.52n, Wayne Rasband, National Institutes of Health, Bethesda, MD, USA) with the ‘Color Pixel Counter’ plugin. The average number of green pixels per cell was measured using a fixed threshold value that was applied consistently across all images.

(c) Flow cytometric analysis of CM-H_2_DCFDA-stained cells

For analysis on a flow cytometer, cells were seeded on a 6-well plate. After trypsinization and centrifugation at 300× *g*, 5 min, the pellets were washed with DPBS and then centrifuged again at 300× *g*, 5 min. The pellets were resuspended in DPBS, and then the suspension was transferred to another tube as a control sample (unstained), and then centrifuged as before. A 100 µL volume of DPBS was added to the control sample after the supernatant was drawn off, and 100 µL of the prepared reagent was added to the test sample; both samples were then incubated for 30 min in an incubator. After incubation, the cells were washed three times with DPBS and finally resuspended in 350 µL of DPBS. CM-H2DCFDA-positive cells were counted on a BD LSRFortessa™ Cell Analyzer (Becton, Dickinson and Company, BD Biosciences, San Jose, Belgium) using an FITC detector.

### 4.4. Senescence Analysis

Cellular senescence was analyzed using the β-galactosidase hydrolysis kit (Senescence Cells Histochemical Staining Kit, Sigma Aldrich, St. Louis, MO, USA) according to the manufacturer’s instructions. The cells were seeded in a 12-well plate, and were first treated with fixation buffer and incubated for 6 min at RT. Next, the cells were treated with staining mixture and incubated overnight at 37 °C. The photos were taken the next day using the Leica DMi1 inverted microscope (Leica Microsystems, Wetzlar, Germany). Color analysis was performed in Fiji using ImageJ Software (version 1.52n, Wayne Rasband, National Institutes of Health, USA) using the ‘Color Pixel Counter’ plugin to measure the total area of green pixels in each image.

### 4.5. Evaluation of Viability and Apoptosis

Cell viability and apoptosis were analyzed using the Muse^®^ Annexin V & Dead Cell Kit (Luminex, Austin, TX, USA), following the manufacturer’s instructions. Briefly, approximately 0.3 × 10^6^ cells were suspended in 100 μL of Annexin V & Dead Cell Reagent. Cells were incubated for 20 min in the dark at RT and then analyzed using the Muse™ Cell Analyzer (EMD Millipore, Temecula, CA, USA). The analysis provided the percentage of live, early apoptotic, late apoptotic, and dead cells.

### 4.6. Evaluation of Cell Proliferation

(a) Immunocytochemical detection of Ki67 expression

The viability of EqASCs was assessed by examining the expression level of the Ki67 cell proliferation marker [48]. Initially, 400 µL of medium containing 0.6 × 10^5^ cells was seeded on coverslips in a 24-well plate. After treating the cells with orientin for 24 h, they were fixed in 4% PFA solution for 20 min at RT. The wells were then washed twice with cold DPBS solution and the cell membranes were next permeabilized using 0.1% Triton X-100 (Sigma-Aldrich, St. Louis, MO, USA) for 5 min at RT, after which they were again washed twice with cold DPBS solution. Removing excess DPBS, the slides were treated with a blocking solution (1% BSA, Sigma-Aldrich, St. Louis, MO, USA, 10% Normal Goat Serum, Abcam, Cambridge, UK, 0.3M Glycine, Sigma-Aldrich, St. Louis, MO, USA, 0.1% Tween^®^ 20, Sigma-Aldrich, St. Louis, MO, USA in DPBS) for 1 h at RT. After blocking, the cells were washed twice with cold DPBS, and after removing the DPBS, Ki67 primary antibody solution (#ab15580, Abcam, Cambridge, UK) was added to the coverslips at a concentration of 0.5 µg/mL (1:250 in DPBS) and incubated overnight at 4 °C. After incubation, the coverslips were washed twice with cold DPBS and a solution of secondary antibody (anti-Rabbit) conjugated with Alexa Fluor 488 fluorochrome (#111-545-003, Jackson ImmunoResearch, Ely, UK) at a dilution of 1:800 in DPBS was added. The coverslips were again incubated for 1 h in the dark at RT. After incubation, the coverslips were rinsed twice with cold DPBS and sealed in 4′,6-Diamidino-2′- phenylindole dihydrochloride (DAPI) mounting medium (Fluoroshield™ with DAPI, Sigma-Aldrich, St. Louis, MO, USA). Preparations were observed and imaged using a confocal laser scanning microscope (Leica DMi8, Leica Microsystems, Wetzlar, Germany) and micrographs processed with the Fiji ImageJ software (Version 1.52n, Wayne Rasband, National Institutes of Health, USA). The mean green pixel area per cell was determined using the same threshold value for all images.

(b) Cell cycle distribution analysis by flow cytometry

The percentage of cells in the G0/G1, S, and G2/M phases of cell cycle was analyzed using the Muse^®^ Cell Cycle Kit (Luminex, Austin, TX, USA). In accordance with the manufacturer’s protocol, approximately 0.3 × 10^6^ cells were suspended in 200 μL of ice cold 70% ethanol and incubated for 3 h at −20 °C. After incubation, the cells were centrifuged at 300× *g* for 5 min and washed once with DPBS. Next, 200 μL of Muse^®^ Cell Cycle Reagent was added to each tube and incubated for 30 min at RT in the dark and then analyzed using the Muse™ Cell Analyzer (EMD Millipore, Temecula, CA, USA).

(c) Clonogenic potential assessed by colony-forming unit (CFU) assay

The colony unit forming (CFU) assay was used to evaluate the clonogenic property of the studied ASCs [49]. Approximately 250 cells were seeded into a 6-well plate and after 24 h they were treated with orientin, as previously described. After 11 days the cells were washed with DPBS and stained with 0.02% pararosaniline solution (Sigma-Aldrich, St. Louis, MO, USA), allowing the colonies to be counted manually based on visual assessment.

(d) Cell migration and interaction asessed by scratch assay

A scratch assay was used to evaluate cell migration and intercellular interactions [48]. Cells were seeded into a 12-well plate and treated with orientin, as previously described. Once a uniform, confluent monolayer was formed, a sterile pipette tip was used to create a scratch across the center of each well, simulating a wound. The distance between the wound edges was measured using the Leica DMi1 inverted microscope (Leica Microsystems, Wetzlar, Germany) at 6- and 24-h post-scratch.

### 4.7. Reverse Transcription Quantitative Real-Time PCR Analysis

Total RNA was isolated using the phenol–chloroform method following cell homogenization in EXTRAzol solution (Blirt, Gdańsk, Poland). The RNA concentration was measured using a nanospectrophotometer (Synergy H1, BioTek^®^ Instruments, Inc., Winooski, VT, USA) at 260 nm. Genomic DNA (gDNA) was digested using the Precision^TM^ DNase Kit (Primerdesign Ltd., Chandler’s Ford, UK). The cDNA was synthesized using the RevertAid RT Reverse Transcription Kit (Thermo Fisher Scientific, Vilnius, Lithuania) in a T100^TM^ Thermal Cycler (Bio-Rad Laboratories, Singapore). qRT-PCR reactions were prepared using the SensiFAST™ SYBR^®^ & Fluorescein Kit (Meridian, Cincinnati, OH, USA) and analyzed in a CFX Connect^TM^ Real-Time PCR Detection System (Bio-Rad Laboratories, Singapore). All procedures were carried out in accordance with the respective manufacturers’ instructions. Primers used in the qRT-PCR were designed using the Primer-BLAST tool provided by the National Center for Biotechnology Information (NCBI), U.S. National Library of Medicine. The relative expression of genes (Table 1) was normalized to GAPDH (glyceraldehydes-3-phosphate, housekeeping gene) expression and calculated using the 2^−∆∆Cq^ method.

### 4.8. Statistical Analyses

The results were presented as mean ± SD. The statistics were performed using GraphPad Prism version 10.5.0 for Windows (GraphPad Software, Boston, MA, USA). The Shapiro–Wilk test was used to confirm the normality of distribution, and the homogeneity of variances was verified using Bartlett’s test. Two-way analysis of variance (ANOVA) was applied, focusing on the main effects only, with ‘health status’ (HE vs. EMS) and ‘treatment’ (CTRL vs. Orientin) as the two independent factors. Tukey’s post hoc test was used for multiple comparisons. All results were expressed as relative values, where the mean value obtained for the untreated healthy control group was set as 1, and the values of the other groups were expressed relative to this reference. The differences were considered as statistically significant at *p* < 0.05.

## 5. Conclusions

The present study suggests that orientin can exert multifaceted cytoprotective and regenerative effects on EqASCs, particularly those derived from horses affected by EMS. Orientin treatment enhanced proliferative activity and favorable modulation of the cell cycle, improved cell viability, reduced apoptosis, and restored both clonogenic and migratory capacity. Importantly, orientin significantly reduced markers of oxidative stress and cellular senescence, two key factors contributing to impaired regenerative function in EMS-associated stromal cells. These findings indicate that orientin’s therapeutic potential lies in mitigating cellular dysfunction associated with metabolic disorders in equine models. However, further studies are needed to fully elucidate the molecular mechanisms underlying orientin’s action, particularly in relation to oxidative stress response pathways and the regulation of senescence. Future research should explore the involvement of key signaling axes such as AMPK, PI3K/Akt, and FOXO, as well as assess orientin’s long-term effects and therapeutic efficacy in vivo. A deeper understanding of these mechanisms may facilitate development of targeted strategies to restore cellular homeostasis and regenerative potential in metabolically impaired tissues.

## Figures and Tables

**Table 1:** Primers used for gene expression analysis.

Gene	Primer	Sequence 5′-3′	Amplicon Length (bp)	Accession No.
*GAPDH*	F: R:	GATGCCCCAATGTTTGTGA AAGCAGGGATGATGTTCTGG	250	NM_001163856.1
*p21*	F: R:	GAAGAGAAACCCCCAGCTCC TGACTGCATCAAACCCCACA	241	XM_023633878.1
*SOD1*	F: R:	CATTCCATCATTGGCCGCAC GAGCGATCCCAATCACACCA	130	NM_001081826.3
*SOD2*	F: R:	GGACAAACCTGAGCCCCAAT TTGGACACCAGCCGATACAG	125	NM_001082517.2
*TP53*	F: R:	TTTCGACATAGCGTGGTGGT CTCAAAGCTGTTCCGTCCCA	180	NM_001202405.1
*BAX*	F: R:	CGAGTGGCAGCTGAGATGTT AAGGAAGTCCAGTGTCCAGC	153	XM_023650076.1
*BCL2*	F: R:	TTCTTTGAGTTCGGTGGGGT GGGCCGTACAGTTCCACAA	164	XM_001490436.4

## Data Availability

The data presented in this study are available on request from the corresponding author.

## Supplementary Materials

The following supporting information can be downloaded at: https://www.mdpi.com/article/10.3390/ijms26146867/s1.

## Abbreviations

The following abbreviations are used in this manuscript:

EMS	Equine Metabolic Syndrome
ASCs	Adipose-Derived Mesenchymal Stromal Cells
SA-β-Gal	Senescence-Associated β-Galactosidase
SASP	Senescence-Associated Secretory Phenotype
ROS	Reactive Oxygen Species
mTOR	Mammalian Target of Rapamycin
FOXO	Forkhead Box O (transcription factors)
TNF-α	Tumor Necrosis Factor Alpha
IL-6	Interleukin 6
MMPs	Matrix Metalloproteinases
SOD	Superoxide Dismutase
GPx	Glutathione Peroxidase
PI3K	Phosphoinositide 3-Kinase
Akt	Protein Kinase B
AMPK	AMP-Activated Protein Kinase
p16^INK4a	Cyclin-Dependent Kinase Inhibitor 2A (also known as p16INK4a)
p21	Cyclin-Dependent Kinase Inhibitor 1A (CDKN1A)
TP53	Tumor Protein P53
CM-H2DCFDA	5-(and-6)-Chloromethyl-2′,7′-Dichlorodihydrofluorescein Diacetate, Acetyl Ester
DAPI	4′,6-Diamidino-2-Phenylindole
