# Supplementary material for: Orientin Reverses Premature Senescence in Equine Adipose Stromal Cells Affected by Equine Metabolic Syndrome Through Oxidative Stress Modulation
Source: Int J Mol Sci. 2025 Jul 17;26(14):6867. doi: 10.3390/ijms26146867 (PMC12295333; doi:10.3390/ijms26146867)
Supplement: Supplementary file 1 [file ijms-26-06867-s001.zip › ijms-3693733-supplementary.pdf]

NonLinear Regression - Global Curve Fitting

Equation: Ligand Binding; sigmoidal dose-response
f = min + (max-min)/(1+10^(logEC50-x))

Global Goodness of Fit:

R	Rsqr	Adj Rsqr	Standard Error of Estimate
0,9815	0,9633	0,9528	0,0308

Analysis of Variance:

	DF	SS	MS
Regression	3	4,1810	1,3937
Residual	7	0,0067	0,0010
Total	10	4,1877	0,4188

Corrected for the mean of the observations:

	DF	SS	MS
Regression	2	0,1745	0,0873
Residual	7	0,0067	0,0010
Total	9	0,1812	0,0201

Statistical Tests:

Normality Test (Shapiro-Wilk) Passed (P = 0,1266)

W Statistic= 0,8789 Significance Level = <0,0001

Constant Variance Test (Spearman Rank Correlation) Passed (P = 0,7849)

Number of Observations = 10
Rsqr = 0,9633
Residual Sum of Squares = 0,0067

Parameter Estimates:

	Coefficient	Std. Error	t	P
min	0,5289	0,0126	42,0240	<0,0001
max	0,8208	0,0178	46,1159	<0,0001
logEC50	59,8875	0,1990	300,9470	<0,0001

Fit Equation Description:
[Variables]
g_sign(p;q) = if(xatymax(p;q)-xatymax(p;max(q)-q)>0;1;-1)
f0_x = col(1)
f0_y = col(2)
f0_reciprocal_y = 1/abs(f0_y)
f0_reciprocal_ysquare = 1/f0_y^2
